# Neurabin in the anterior cingulate cortex regulates anxiety-like behavior in adult mice

**DOI:** 10.1186/1756-6606-4-6

**Published:** 2011-01-19

**Authors:** Susan S Kim, Hansen Wang, Xiang-Yao Li, Tao Chen, Valentina Mercaldo, Giannina Descalzi, Long-Jun Wu, Min Zhuo

**Affiliations:** 1 Department of Physiology, Faculty of Medicine, University of Toronto Centre for the Study of Pain, 1 King's College Circle, Toronto, ON, Canada; 2 Department of Brain and Cognitive Sciences, College of Natural Sciences, Seoul National University, Seoul 151-746, Korea

## Abstract

Affective disorders, which include anxiety and depression, are highly prevalent and have overwhelming emotional and physical symptoms. Despite human brain imaging studies, which have implicated the prefrontal cortex including the anterior cingulate cortex (ACC), little is known about the ACC in anxiety disorders. Here we show that the ACC does modulate anxiety-like behavior in adult mice, and have identified a protein that is critical for this modulation. Absence of neurabin, a cytoskeletal protein, resulted in reduced anxiety-like behavior and increased depression-like behavior. Selective inhibition of neurabin in the ACC reproduced the anxiety but not the depression phenotype. Furthermore, loss of neurabin increased the presynaptic release of glutamate and cingulate neuronal excitability. These findings reveal novel roles of the ACC in anxiety disorders, and provide a new therapeutic target for the treatment of anxiety disorders.

## Introduction

Affective disorders are among the most common psychiatric diagnoses [1]. Selective Serotonin Reuptake Inhibitors (SSRIs) are the most commonly prescribed drugs for such mood disorders, but very little is known regarding the molecular mechanisms and the specific neural circuits that underlie both the disorders and the drugs' effects [2]. Additionally, while treatments for affective disorders are effective, many patients are left with residual symptoms or experience side effects that limit their adherence to prescribed regimens [3]. Although numerous human brain imaging studies have implicated the prefrontal cortex (PFC) including the ACC in affective disorders [4-6], the exact role of the ACC in affective disorders still remains unknown.

Neurabin is a cytoskeletal protein found both in dendritic spines and axon terminals [7]. Neurabin interacts directly with the filamentous actin cytoskeleton [8,9]. In addition to the actin-binding domain, neurabin contains several other domains critical for their function, such as protein phosphatase 1(PP1)-binding motif [10,11]. Previous studies in the hippocampus have found that neurabin contributes to the regulation of glutamate AMPA receptor functions and its related plasticity [12]. Additionally, numerous drugs affecting anxiety affect AMPA receptor-mediated excitatory transmission in the PFC [13], and antagonism of AMPA receptors have been found to reduce anxiety-like behaviors [14,15]. Studies have also found that synaptic transmission and plasticity in the ACC is critical for pain, fear, and learning and memory [16-24], and recently, it was found that neurons in the ACC form local excitatory and inhibitory connections [25]. In the ACC, excitatory synaptic transmission is mediated by glutamate and GABA mediates inhibitory synaptic transmission. As benzodiazepines, which are clinically used to treat anxiety disorders, promote binding of GABA to GABA_A _receptor, these connections in the ACC could be essential in modulating anxiety-like behaviors.

Here, we demonstrate that the injections of muscimol, a potent, selective GABA_A _receptor agonist, as well as midazolam, a clinically used benzodiazepine, into the ACC reduced anxiety-like behaviors. Neurabin knockout (KO) mice had reduced anxiety-like behavior but increased depression-like behavior. Selective inhibition of neurabin in the ACC using siRNA reproduced the anxiety-like behavior phenotype but did not affect the depression-like behavior. Furthermore, deletion or reduction of neurabin increased the presynaptic release of glutamate and cingulate neuronal excitability. This study demonstrates the role of the ACC in affective disorders, and that neurabin may be a possible new therapeutic target for the treatment of anxiety disorders.

## Materials and methods

### Animals

Adult male mice (8-12 weeks old) were used for all experiments. All mice were maintained on a 12 h light/dark cycle with food and water provided *ad libitum*. All protocols used were approved by The Animal Care and Use Committee at the University of Toronto.

### Behavioral tests

#### Elevated plus maze

The apparatus consisted of two opposing open and closed arms. For each test, the animal was placed in the centre square and allowed to move freely for 5 min. The number of entries and time spent in each arm were recorded.

#### Open field

To record horizontal activity, Activity Monitor system from Med Associates (St. Albans, VT) was used. Briefly, this system used paired sets of photo beams to detect movement in the open field and movement was recorded as beam breaks. The open field was placed inside an isolation chamber with dim illumination and a fan. Each subject was placed in the centre of the open field and activity was measured for 30 minutes.

#### Light/dark test

The apparatus consisted of a rectangular Plexiglas box (44 × 8.5 × 25 cm) equally divided into a light and dark compartment that was separated by a door. Each animal was placed in the light compartment and was allowed 10 sec to explore, after which the door to the dark compartment was opened. The time spent in both compartments was recorded for 10 min.

#### Elevated emergence task

The apparatus consisted of a rectangular Plexiglas floor equally divided into opaque and clear sections, with the opaque section surrounded on three sides by high walls, resting on an elevated platform, while clear section was not enclosed and suspended over the floor from the elevated platform. For each test, the animal was placed in the opaque section and was allowed to move freely for 5 min. The latency to cross into the clear floor and time spent were recorded.

#### Forced swim test

Mice were placed in a transparent plastic tub containing water (23-25°C). Each mouse was tested for 6 minutes. The mouse was allowed to swim freely for 2 min. Time spent immobile (aside from small maintenance movements) was recorded during the last 4 minutes. After the test, the animals were allowed to dry in a heated cage before being returned to the home cage. The water was changed after each mouse.

### Western blot

Briefly, cultured cortical neurons were harvested and homogenized in lysis buffer. Electrophoresis of equal amounts of total protein was run on SDS-polyacrylamide gels. Separated proteins were transferred to polyvinylidene fluoride membranes overnight at 4°C. Membranes were probed with primary antibodies against neurabin (Millipore Corporation, California) and actin (Sigma, MO) overnight at 4°C. The membranes were then incubated with horseradish peroxidase-coupled secondary antibody for 2 h at room temperature followed by enhanced chemiluminescence (ECL) detection using Western Lightning Chemiluminescence Reagent Plus (Perkin Elmer Life sciences, MA). The density of immunoblots was measured with NIH ImageJ software.

### Brain cannulation and microinjection

Mice were anesthetized with i.p. injection of a mixture of xylazine (10 mg/kg) and ketamine (130 mg/kg). The fur above the skull was shaved and the skin cleaned with alcohol, Triadine, then alcohol. The head of the mouse was placed in a stereotaxic device and lubricant applied to the eyes. An incision was made over the skull and the surface exposed. A 24 gauge guide cannula was implanted bilaterally into the ACC. Dental cement was used to keep the cannula in place and sterile silk sutures were used to close the skin. Mice were injected with 1.0 ml sterile saline (i.p.) for hydration. Animals were allowed to recover in a chamber containing a recirculating water blanket until their righting reflex returned; they were then placed in a clean cage and given moistened food. Ketoprofen (0.5 mg/ml) was given intraoperatively as an analgesic. The animal was given at least one week to fully recover. For microinjection, a 30 gauge injection cannula, which is 0.8 mm lower than the guide, were used for drug infusion, 0.5 ul delivered over 90 sec. Behavioral responses were measured 20 min after microinjection.

### Primary cortical neuron culture

Cortical neurons were prepared from postnatal day 0 (P0) mice using methods as described previously [26,27]. The cortices were dissected, minced, and trypsinized for 15 min using 0.125% trypsin (Invitrogen, CA). Cultures were grown in Neurobasal-A medium supplemented with B27 and 2 mM GlutaMax (Invitrogen, CA) and incubated at 37°C in 95% air, 5% CO2 with 95% humidity. Cultures were transfected with neurabin or control siRNA using Transfection reagent for siRNA (Invitrogen, CA) at DIV 10. The expression of neurabin was detected 40 hours later by Western blot.

### Electrophysiology

Coronal brain slices (300 μm) containing the ACC from six- to eight-week-old male mice were prepared using standard methods [22]. Slices were transferred to a submerged recovery chamber with oxygenated (95% O_2_and 5% CO_2_) artificial cerebrospinal fluid (ACSF) containing (in mM): 124 NaCl, 2.5 KCl, 2 CaCl_2_, 2 MgSO_4_, 25 NaHCO_3_, 1 NaH_2_PO_4_, 10 glucose at room temperature for at least 1 hour.

All electrophysiological experiments were performed at room temperature. An Olympus BX51WI microscope (Tokyo, Japan) with infrared DIC optics was used for visualization of whole-cell patch clamp recording. Excitatory postsynaptic currents (EPSCs) were recorded from layer II/III neurons with an Axon 200 B amplifier (Molecular devices, CA) and the stimulations were delivered by a bipolar tungsten stimulating electrode placed in layer V of the ACC slices. EPSCs were induced by repetitive stimulations (duration is 200 μs, intensity is adjusted to induce EPSCs with amplitude of 50-100 pA at 0.05 Hz and neurons were voltage clamped at -70 mV. The recording pipettes (3-5 MΩ) were filled with solution containing (mM): 120 K-gluconate, 5 NaCl, 1 MgCl_2_, 0.2 EGTA, 10 HEPES, 2 Mg-ATP, 0.1 Na_3_-GTP and 10 phosphocreatine disodium salt (adjusted to pH 7.2 with KOH). Picrotoxin (100 μM) was always present to block GABA_A_receptor-mediated inhibitory currents and monitored throughout the synaptic currents. In the mEPSC and mIPSC experiments, 1 μM TTX were perfused into the ACSF to blocking the activities of sodium currents. Access resistance was 15-30 MΩ and was monitored throughout the experiment. Data were discarded if access resistance changed more than 15% during an experiment.

### siRNA preparation and microinjection

Stealth neurabin-targeted siRNA or siRNA negative control (Invitrogen, CA) and Invivofectamine Reagent (Invitrogen, CA) were gently mixed together then incubated for 30 min at room temperature in an orbital shaker. 5% glucose was added, and the mixture was transferred into an Amicon Ultra-4 Centrifugal Filter Device (Ultracel - 100 k; Millipore, MA) and centrifuged for 1 hour.

Mice were anesthetized with i.p. injection of a mixture of xylazine (10 mg/kg) and ketamine (130 mg/kg). A 30 gauge needle was lowered into the ACC (AP 0.7 mm; ML 0.3 mm; VD 1.75 mm) and the prepared siRNA was injected. Mice were injected with 1.0 ml sterile saline (i.p.) for hydration. Ketoprofen (0.5 mg/ml) was given intraoperatively as an analgesic.

### Data analysis

Statistical comparisons were made using the Student's t-test or two way ANOVA to identify significant differences. All data are expressed as mean value ± standard error of the mean. In all cases, *p *< 0.05 was considered statistically significant.

## Results

### The ACC is critical for anxiety- and depression-like behaviors

**Figure 1:**
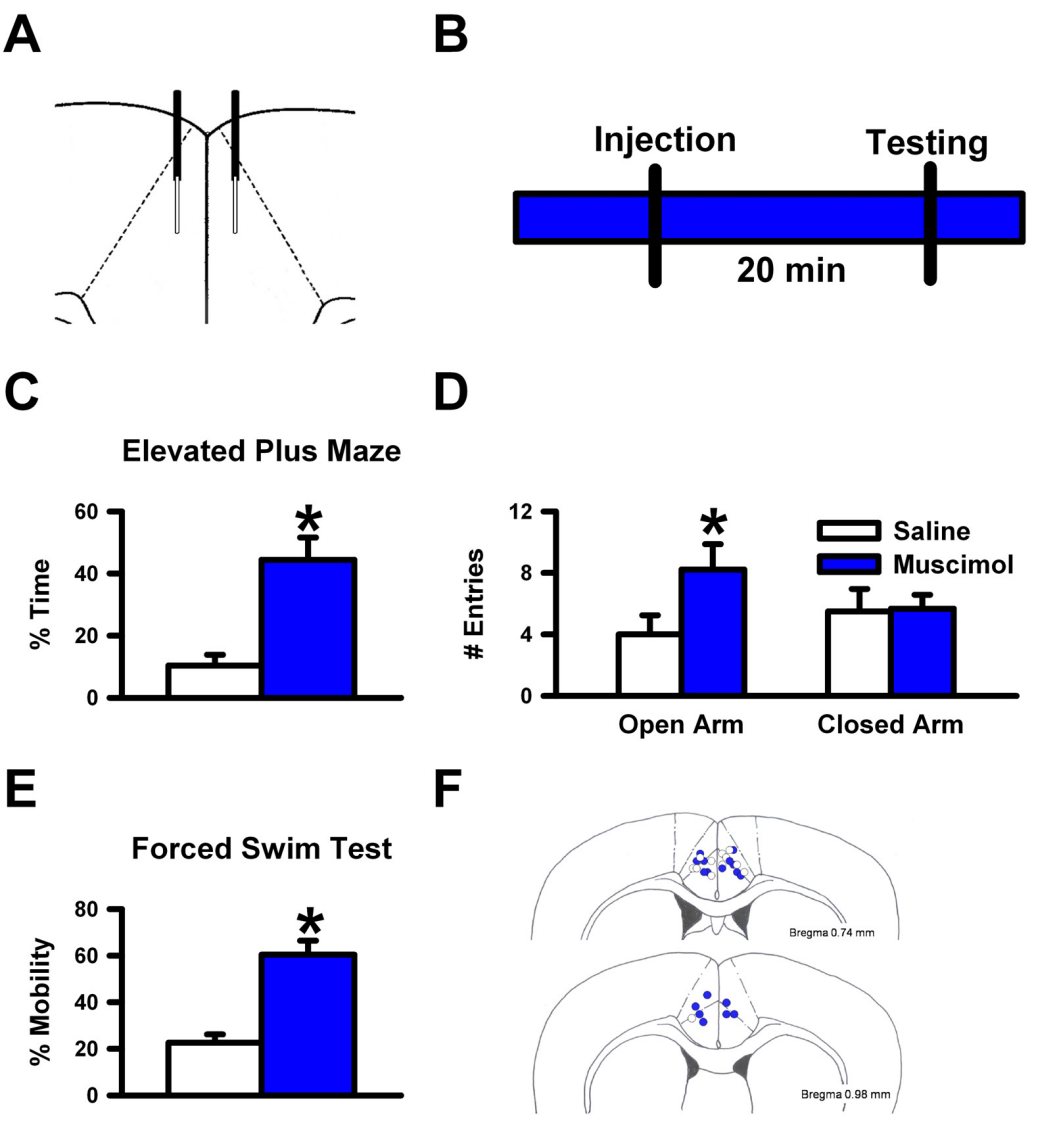
**The ACC is critical for anxiety- and depression-like behaviors**. ***A***, Model showing the cannulation and microinjection into the ACC. ***B***, Diagram showing the timeline of experiment. ***C***, Percent of time in the open arms of the elevated plus maze. ***D***, Number of entries into the open and closed arms of the elevated plus maze. ***E***, Percent of active mobility in the forced swim test. ***F***, Microinjection sites in the ACC.

**Figure 2:**
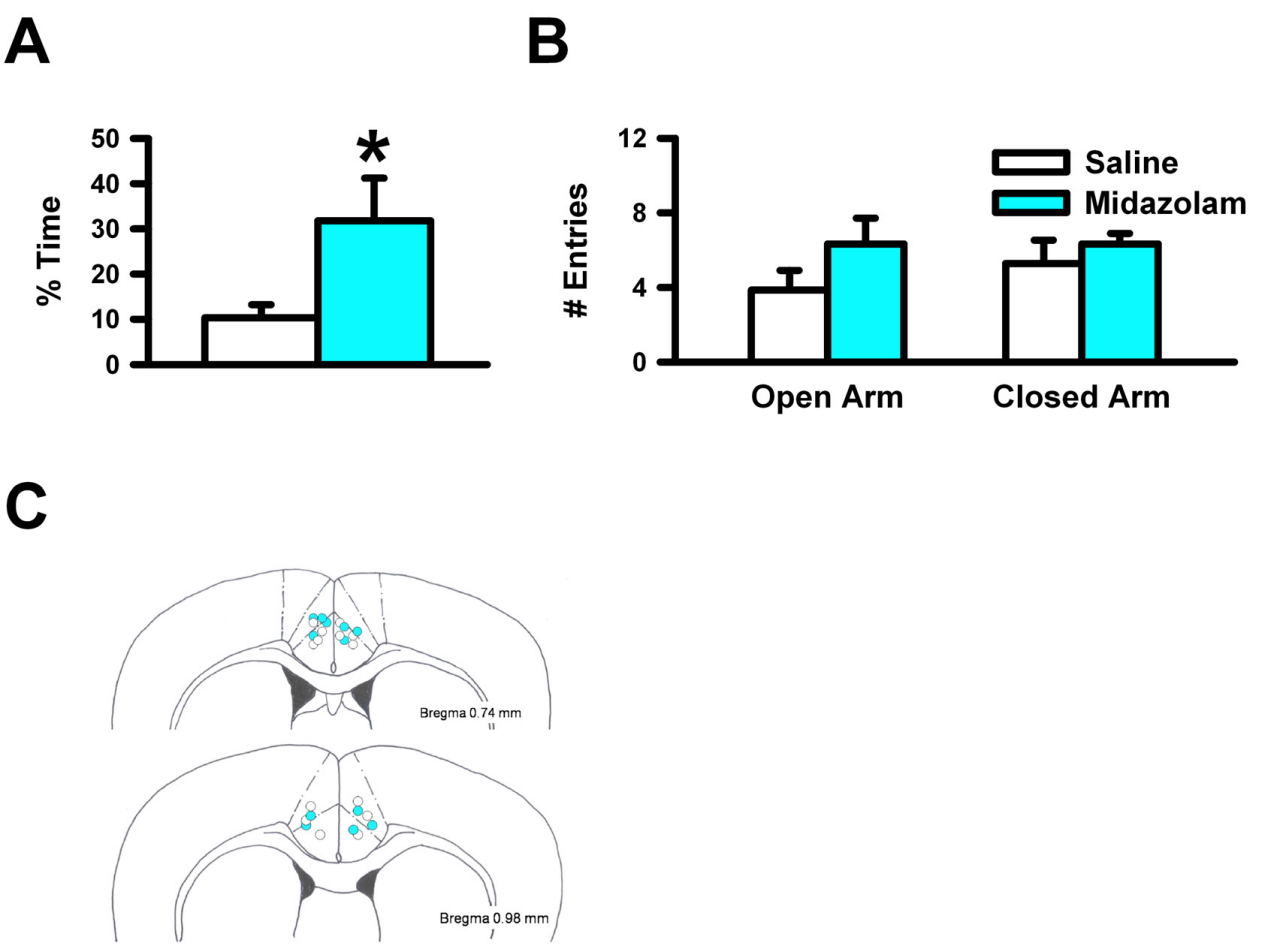
**Microinjection of a clinically used benzodiazepine into the ACC reduced anxiety-like behavior**. ***A***, Percent of time in the open arms of the elevated plus maze. ***B***, Number of entries into the open and closed arms of the elevated plus maze. ***C***, Microinjection sites in the ACC.

To determine the role of the ACC in anxiety, we microinjected muscimol, a potent, selective GABA_A _receptor agonist, into the ACC of C57BL/6 mice, then tested them on the elevated plus maze. Muscimol-injected mice spent significantly more time (Figure 1C; n = 9 for muscimol, n = 6 for saline, * *p *< 0.05) and made more entries into the open arms (Figure 1D) of the elevated plus maze than the saline-injected mice, suggesting that the ACC was critical for anxiety-like behaviors. There were no differences between groups for closed arm entries (Figure 1D), indicating that the phenotypic differences were not due to hyperactivity. Next, we microinjected into the ACC midazolam, a short-acting benzodiazepine that has potent anxiolytic properties. Similarly to mice microinjected with muscimol, midazolam-injected mice spent significantly more time in the open arms of the elevated plus maze than the saline-injected mice (Figure 2A; n = 6 for midazolam, n = 7 for saline, * *p *< 0.05), suggesting that injecting a clinically used benzodiazepine into the ACC was sufficient enough to reduce anxiety-like behavior. There were no differences between groups for closed arm entries (Figure 2B). Additionally, since anxiety and depression are often comorbid in patients, we tested for depression-related behavior, as measured by the forced swim test. The forced swim test paradigm reflects the behavioral response to inescapable stress, not conflict, and is sensitive to antidepressant but not anxiolytic treatments [28,29]. Rodents usually struggle to escape from these situations, interspersed with periods of immobility that has been interpreted as "behavioral despair" [30]. When we used the forced swim test to assess mice microinjected with muscimol, we found decreased immobility when compared to the controls (Figure 1E; n = 8 for muscimol, n = 6 for saline, * *p *< 0.05). The data suggest that the ACC is critical for both anxiety- and depression-like behaviors.

### Neurabin modulates anxiety-like behavior of the elevated plus maze

**Figure 3:**
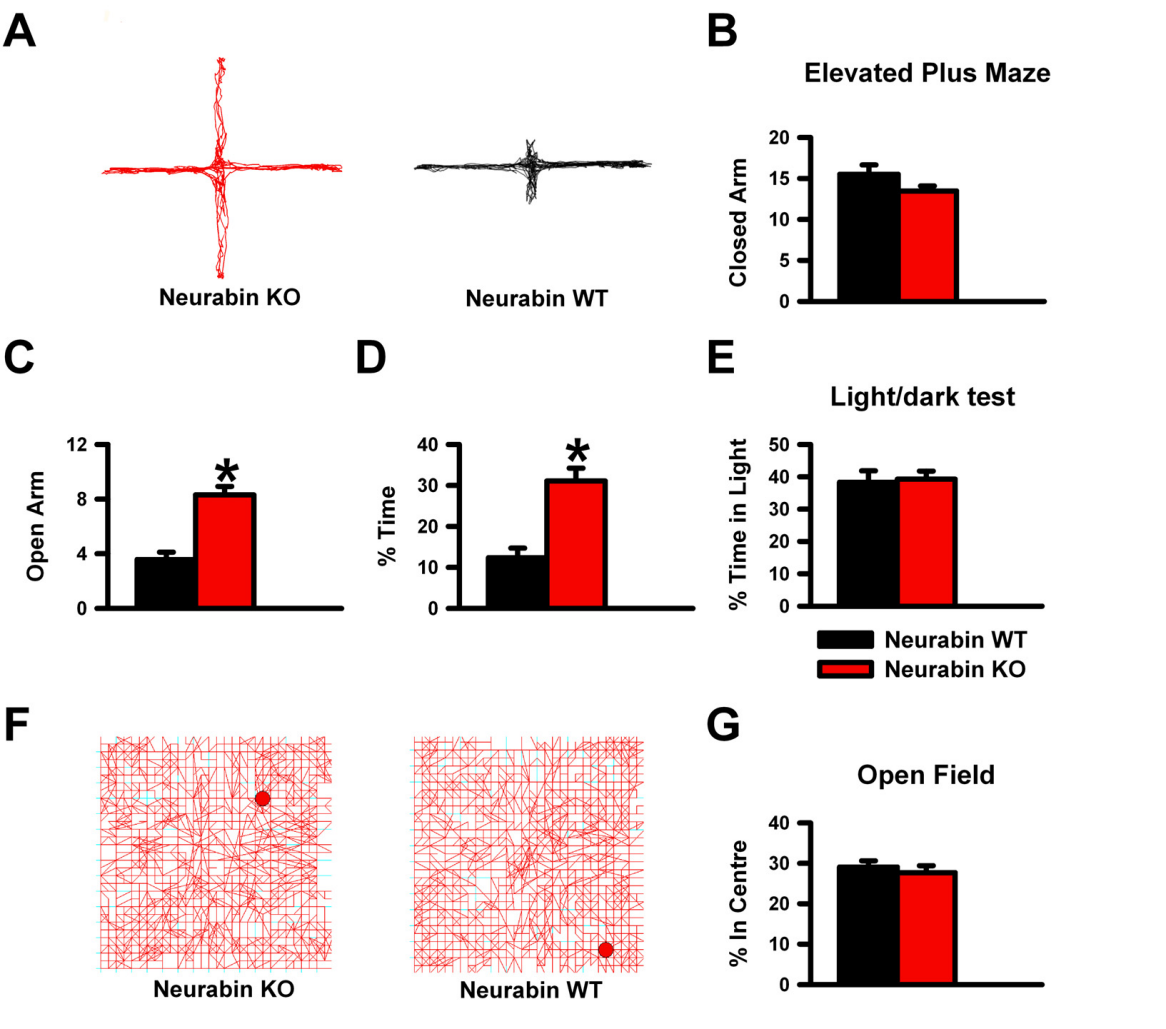
**Neurabin modulates anxiety-like behavior of the elevated plus maze**. ***A***, Sample traces in the elevated plus maze. ***B***, Number of entries into the closed arms of the elevated plus maze. ***C***, Number of open arm entries. ***D***, Percent of time in the open arms. ***E***, Percent of time in the light box of the light/dark test. ***F***, Sample traces in the open field. ***G,***Percent path length in the centre of the open field.

**Figure 4:**
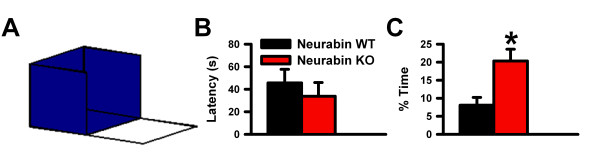
**Neurabin KO mice in the elevated emergence task**. ***A***, Model of the elevated emergence task. ***B***, Latency to cross in the elevated emergence task. ***C***, Percent of time spent in the clear compartment of the elevated emergence task.

Neurabin is a synaptic protein found in both presynaptic terminals and dendritic spines of neurons in the prefrontal cortex [7]. Since neurabin has previously been found to contribute to the regulation of glutamate AMPA receptor functions and its related plasticity in the hippocampus [12], and antagonism of AMPA receptors have been found to produce anxiolytic-like effects [14,15], we hypothesized that inhibition of neurabin may affect anxiety-like behavior. To that end, we examined neurabin knockout (KO) mice in several anxiety paradigms: elevated plus maze, open field, and light/dark test. Neurabin KO mice made significantly more entries into the open arms and spent more time in the open arms of the elevated plus maze than the wildtype mice (Figures 3C and 3D; n = 12 for neurabin KO, n = 6 for neurabin WT, * *p *< 0.05), suggesting that absence of neurabin reduced anxiety-like behaviors. Figure 3A shows sample traces of neurabin KO and WT mice on the elevated plus maze. As well, closed arm entries were comparable between genotypes (Figure 3B), indicating that the increased activity of the neurabin KO mice in the open arms was not due to hyperactivity. When we examined the behavior of neurabin KO mice in two other conflict paradigms, the open field and the light/dark test, we found that exploration of the centre of the open field (as a percentage of total exploratory activity) (Figures 3F and 3G; n = 8 for neurabin KO, n = 8 for WT) as well as the percentage of total time spent in the light compartment during the light/dark test (Figure 3E; n = 10 for neurabin KO, n = 6 for WT) were comparable between genotypes. These results were somewhat surprising given that the open field and light/dark test are also tests of conflict between a rodent's tendencies versus aversive properties [31]. Therefore, to determine if neurabin KO mice are particularly insensitive to the effects of elevation, we devised an elevated emergence task. The apparatus (Figure 4A) consisted of a rectangular Plexiglas floor equally divided into opaque and clear compartments, with the opaque compartment surrounded on three sides by high walls and resting on an elevated platform. The clear compartment was not enclosed and extended out from the platform, suspended over the floor so that the high elevation was starkly apparent. The latency to cross from the dark to the clear compartment, as well as the time spent in the clear compartment were recorded. While the latency to cross did not differ between genotypes (Figure 4B), neurabin KO mice spent significantly more time in the clear compartment than the WT mice (Figure 4C; n = 5 for neurabin KO, n = 5 for WT, * *p *< 0.05). In order to ensure neurabin KO mice did not have problems with visual acuity and depth perception, we examined the mice on the visual cliff test, as it measures gross visual ability [32,33]. We found that the "positive" safe responses remained comparable between groups (71.67 ± 4.77% for neurabin KO, 70.00 ± 8.37% for neurabin WT; n = 6 for KO, n = 5 for WT). Therefore, the data suggest that absence of neurabin could result in reduced sensitivity to the effects of elevation.

### Injecting neurabin siRNA into the ACC reduced anxiety-like behavior

**Figure 5:**
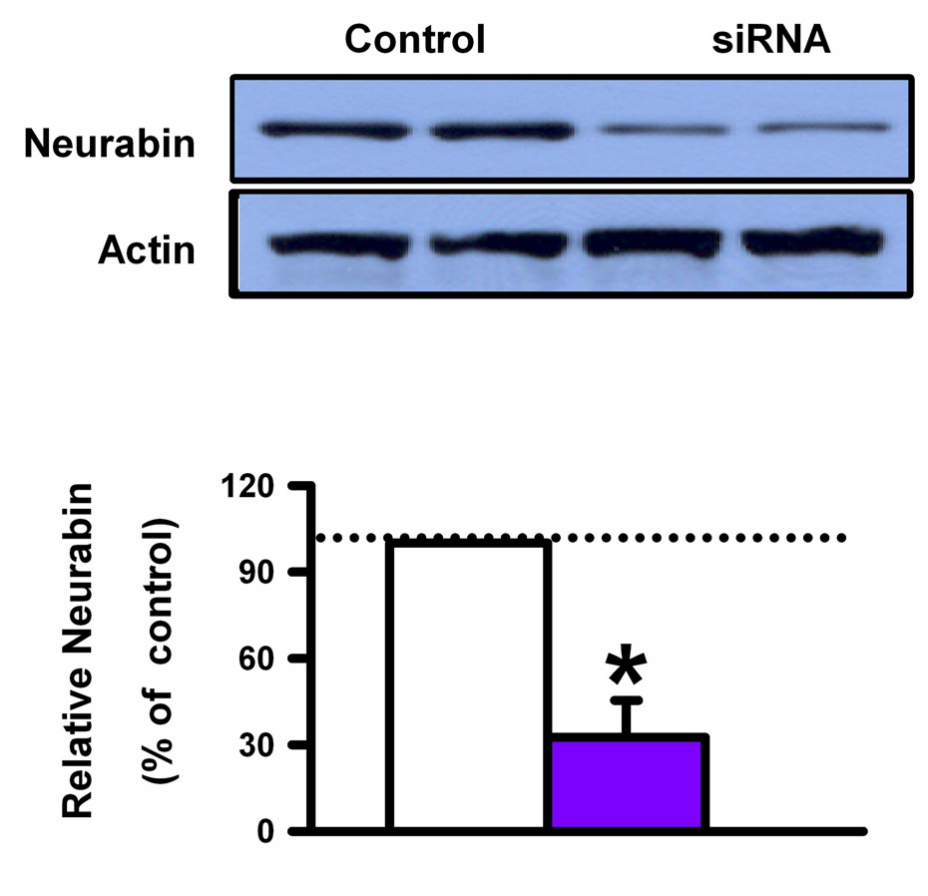
**Neurabin siRNA in primary cortical neuron culture**. ***A***, Reduction of relative neurabin levels in primary cortical neuron culture by neurabin siRNA.

It is difficult to determine the precise regions of the brain that contribute to anxiety in neurabin KO mice since the gene is deleted globally. Furthermore, potential developmental defects or compensatory changes cannot be ruled out. Therefore, using focal application of neurabin siRNA, we investigated whether the inhibition of neurabin expression in the ACC would modulate anxiety-like behavior. First, to confirm the effectiveness of the siRNA, primary cortical neuron culture at DIV 10 was transfected with siRNA negative control or neurabin siRNA (Invitrogen, CA). The expression of neurabin was then measured by Western blot at 48 h after transfection. We found that neurabin siRNA reduced the relative neurabin levels by approximately 70% (Figure 5A). We also saw reduction in neurabin levels when mice were given injections of neurabin siRNA into the ACC for 3 consecutive days (data not shown).

**Figure 6:**
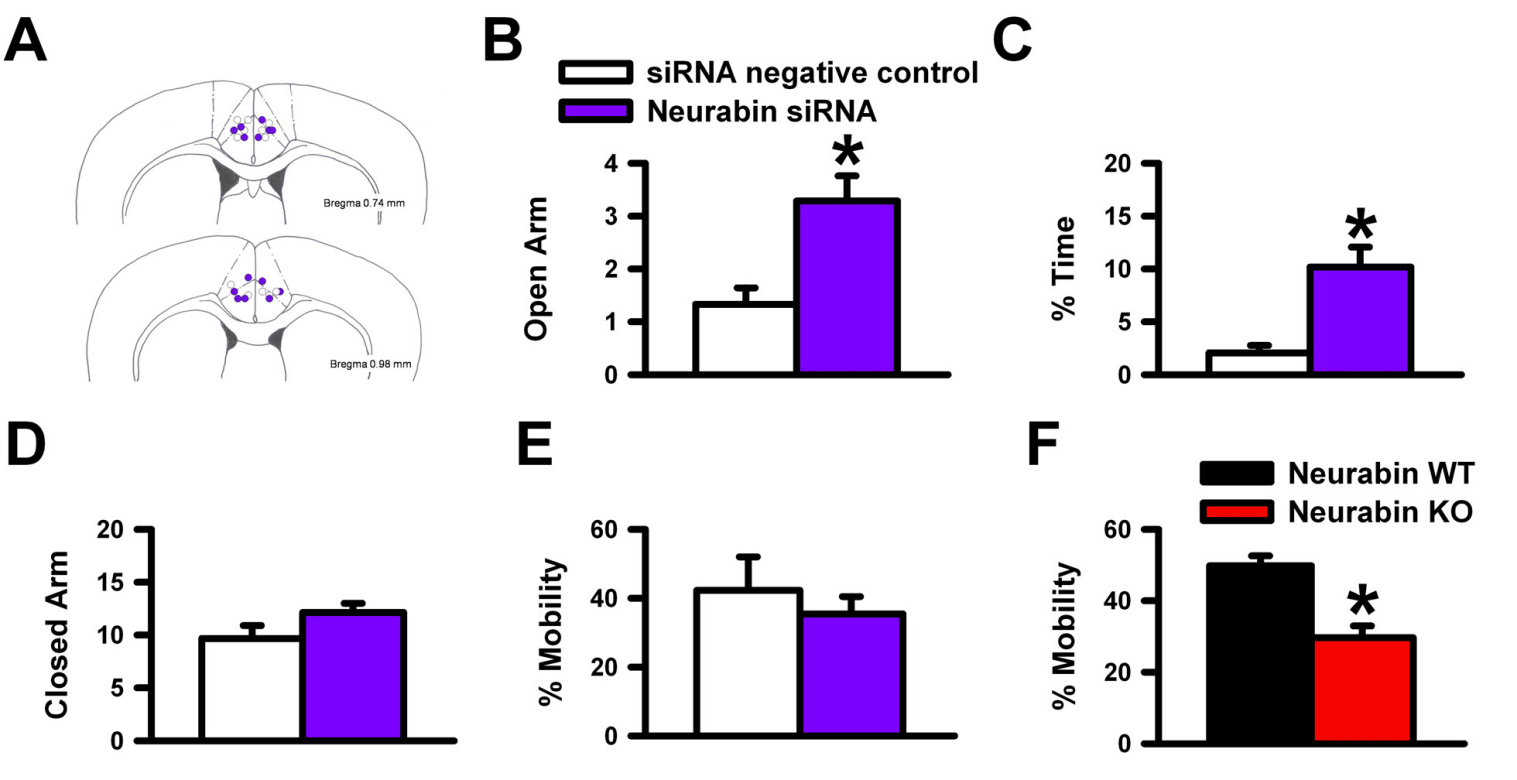
**Microinjectin of neurabin siRNA into the ACC reduced anxiety-like behavior**. ***A***, Injection sites in the ACC. ***B***, Number of entries into the open arms of the elevated plus maze. ***C***, Percent of time in the open arms. ***D***, Number of closed arm entries. ***F***, Percent of active mobility in the forced swim test. ***G***, Percent of active mobility in the forced swim test in neurabin KO and WT mice.

Neurabin siRNA or negative control was then bilaterally injected into the ACC of C57BL/6 mice using Invivofectamine (Invitrogen, CA), a lipid-based transfection reagent. Second or third day following surgery, the mice were tested on the elevated plus maze. While closed arm entries were comparable between groups (Figure 6D), we found that the mice injected with neurabin siRNA made significantly more entries into the open arms and spent more time in the open arms of the elevated plus maze than the siRNA negative control-injected mice (Figures 6B and 6C; n = 7 for neurabin siRNA, n = 6 for siRNA negative control, * *p *< 0.05), suggesting that reduction of neurabin in the ACC was sufficient enough to modulate anxiety-like behaviors. When we used the forced swim test to assess neurabin KO mice, we found increased immobility when compared to the WT mice (Figure 6F; n = 9 for neurabin KO, n = 9 for neurabin WT, * *p *< 0.05), indicating that the loss of neurabin may have increased behavioral despair. Interestingly, when neurabin siRNA was injected into the ACC, there were no significant differences in the forced swim test between groups (Figure 6E; n = 10 for neurabin siRNA, n = 7 for siRNA negative control). These findings demonstrate that neurabin in the ACC may not be required to modulate depression-related behavior.

### Deletion of neurabin increased the presynaptic transmitter release and neuronal excitability in the ACC

**Figure 7:**
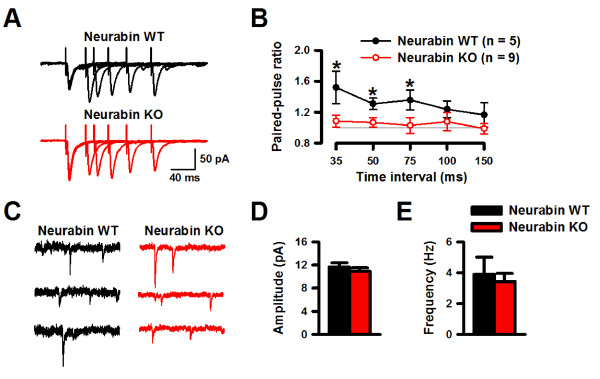
**Deletion of neurabin increased the presynaptic transmitter release and neuron excitability in the ACC**. ***A***, Sample PPF traces from neurabin KO and WT mice. ***B***, Neurabin KO and WT PPF of AMPAR-mediated currents. ***C***, Sample mEPSC recordings from neurabin KO and WT mice. ***D***, Amplitude of mEPSCs. ***E***, Frequency of mEPSCs.

**Figure 8:**
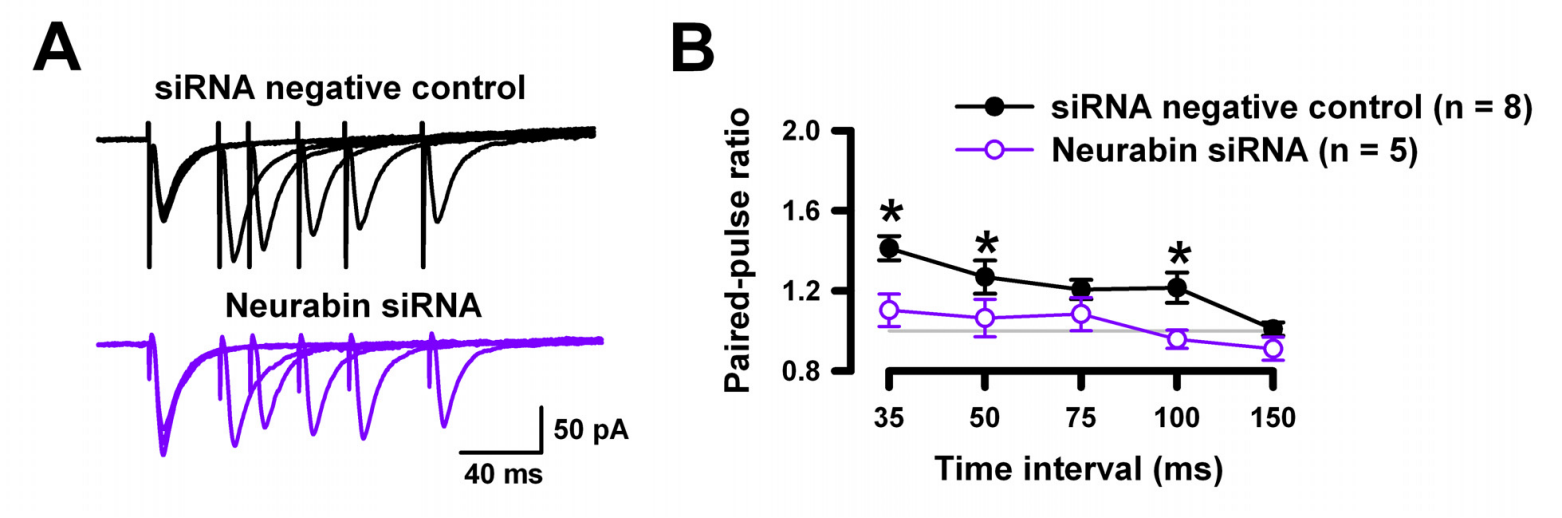
**Injecting neurabin siRNA into the ACC also increased the presynaptic transmitter release and neuron excitability in the ACC**. ***A***, Sample PPF traces from neurabin siRNA and siRNA negative control mice. ***B***, PPF from neurabin siRNA and siRNA negative control mice.

Finally, we wanted to investigate the potential roles of neurabin in the excitatory transmission within the ACC. We found that PPF was significantly reduced in neurabin KO mice when compared to the WT mice (Figures 7A and 7B), suggesting that presynaptic glutamate release was increased in the ACC in neurabin KO mice. Similar results were found in the ACC of mice injected with neurabin siRNA (Figures 8A and 8B). To determine whether the basic synaptic transmission was affected by the absence of neurabin, we further examined the mEPSC in the ACC neurons of neurabin KO and WT mice. Neither the amplitudes nor the frequency of mEPSC (Figures 7C-E) changed between genotypes, indicating that neurabin may contribute to the evoked synaptic responses in the ACC.

## Discussion

Numerous human brain imaging studies have implicated the ACC, an area long known to be engaged in both cognitive and emotional processing [34,35], in affective disorders [4-6]. However, lesion studies have not reflected the brain imaging data [36]. Additionally, few animal studies have examined the role of the ACC in affective disorders, and similar to the human studies, produced conflicting results [37]. Therefore, the exact role of the ACC in affective disorders still remains to be determined.

The present findings are the first, to our knowledge, to show the importance of neurabin in anxiety- and depression-related behaviors. We found that neurabin KO mice showed reduced anxiety-like behaviors in the elevated plus maze. However, when tested on two other conflict paradigms, the open field and the light/dark test, the results remained comparable between genotypes, suggesting that neurabin selectively modulates the anxiety-like behaviors of the elevated plus maze. Next, neurabin-targeted siRNA was injected into the ACC to see if anxiety-like behavior would still be affected without the complete absence of neurabin. Two to three days following injection, the mice were tested on the elevated plus maze, and similarly to neurabin KO mice, also had reduction in anxiety-like behavior, indicating that reducing the level of neurabin in the ACC was sufficient enough for the modulation of anxiety-like behaviors.

As anxiety and depression are often comorbid, forced swim test was used to assess the depression-related behavior of neurabin KO mice. Compared to the WT, neurabin KO mice had increased immobility, suggesting that neurabin KO mice have increased behavioral despair. The result is somewhat surprising, as anxiety and depression have been suggested to share similar genetic vulnerabilities [38,39] and neuropharmacology, supported by findings that SSRIs are effective treatments for both depression and a number of anxiety disorders [40]. That neurabin appears to act in opposing fashion in anxiety- and depression-related behaviors does not negate this theory, as neurabin is critical for the modulation of both, since the absence of neurabin alters the anxiety- and depression-related behaviors. However, neurabin clearly affects both behaviors in different ways, possibly through different neurocircuitries that are specific for anxiety or depression. Interestingly, when neurabin-targeted siRNA was injected into the ACC, there were no significant differences in depression-like behavior between groups, suggesting that neurabin in the ACC may not be required to modulate depression-related behaviors. However, anxiety-related behavior was still reduced by a partial loss of neurabin. This lends support to the idea that neurabin modulates anxiety and depression, but through different neurocircuitries that is specific for one behavior or other. It may also be possible that the reduction in PP1 levels observed by Allen et al. [10] in neurabin KO mice could be absent in mice injected with neurabin-targeted siRNA, and that it may be PP1 activity which is critical for depression-like behavior.

We have also shown that the ACC is indeed critical for anxiety- and depression-like behaviors in mice. Using microinjections of muscimol, a potent, selective GABA_A _receptor agonist, we found that in adult mice, the ACC is critical for anxiety-like behaviors. Mice that had been given microinjections of muscimol into the ACC had reduced anxiety-like behaviors when compared to the mice injected with saline. As well, microinjections of midazolam, a short-acting benzodiazepine, produced similar results, indicating that application of a clinically used anxiolytic into the ACC was sufficient enough to reduce anxiety-like behavior. Additionally, muscimol-injected mice also had reduced depression-like behavior on the forced swim test. However, the closed arm entries and total arm entries remained comparable to controls, suggesting that the phenotypic differences weren't due to hyperactivity. The data, therefore, suggest that the ACC is critical for anxiety- and depression-like behaviors. The work by Bissiere et al. [37], which found that the rostral ACC does not appear to modulate the anxiety-related behavior of rats, doesn't necessarily contradict this current study. As noted by the authors, the area lesioned had selective ipsilateral projections to and from the basolateral amygdala, which previous studies have shown isn't required for the elevated plus maze [41,42]. Additionally, the data from Bissiere et al. show relatively high baseline anxiety, indicating that a possible floor effect may mask potential anxiogenic effects in the elevated plus maze [37]. Furthermore, lesions affect neurons, passing nerve fibers and local glial cells in a non-selective manner, which could have affected the findings.

Our data suggests that neurabin in the ACC is critical for the modulation of the anxiety-like behavior (particularly with respect to elevation) but not depression-like behavior. However, we cannot discount the fact that the elevated plus maze and the elevated emergence task also contain aversive open spaces, even more so than the open field, which is enclosed in a sound-attenuating chamber. Additional tests will need to be devised and conducted to test solely for elevation, and to further determine the downstream effectors of neurabin and their role in affective disorders. In sum, our findings of the requirement of neurabin in anxiety-like behaviors provide a potential novel molecular target for designing new drug treatments for anxiety disorders.

## Competing interests

The authors declare that they have no competing interests.

## Authors' contributions

SSK participated in the design of the study, carried out implantations and injections, behavior experiments and drafted the manuscript. HW carried out siRNA preparations, neuron culture, and participated in running Western blots. XL, TC and LW carried out the electrophysiology experiments. VM and GD participated in running Western blots. MZ conceived of the study, and participated in its design and coordination. All authors read and approved the final manuscript.
